# Crystal structure of *catena*-poly[silver(I)-μ-l-valinato-κ^2^ *N*:*O*]

**DOI:** 10.1107/S2056989017001815

**Published:** 2017-02-14

**Authors:** Yoshitaka Takagi, Youhei Okamoto, Chisato Inoue, Noriko Chikaraishi Kasuga, Kenji Nomiya

**Affiliations:** a Department of Chemistry, Faculty of Science, Kanagawa University, Tsuchiya, Hiratsuka, Kanagawa 259-1293, Japan

**Keywords:** silver(I) complex with amino acid, Ag⋯Ag inter­action, coordination polymer, hydrogen bonding, crystal structure

## Abstract

The reaction of Ag_2_O with l-valine (l-Hval, C_5_H_11_NO_2_) in a 1:2 molar ratio in water, followed by vapour diffusion, afforded a coordination polymer of the title compound, [Ag(C_5_H_10_NO_2_)]_*n*_, with N—Ag—O repeat units, which is classified as a type III silver(I) complex with amino acid ligands. The asymmetric unit consists of two independent units of [Ag(l-val)]. In the crystal, the polymeric chains run along [101], and neighbouring chains are linked *via* a weak Ag⋯Ag inter­action and N—H⋯O hydrogen bonds. The title complex exhibited anti­microbial activity against selected bacteria (*Escherichia coli*, *Bacillus subtilis*, *Staphylococcous aureus* and *Psedomonas aeruginosa*).

## Abstract 2

The reaction of Ag_2_O with l-valine in a 1:2 molar ratio in water, followed by vapour diffusion, afforded polymeric N—Ag—O repeated units of the title silver(I) complex. It shows a weak Ag⋯Ag inter­action and hydrogen bonds between amino groups and carboxyl­ates.

## Chemical context   

Silver(I) complexes with amino acid ligands have been of inter­est not only due to their numerous medicinal applications but also as model protein–silver(I) inter­action compounds (Banti & Hadjikakou, 2013 ▸; Eckhardt *et al.*, 2013 ▸). Aside from S-containing amino acids, such as cysteine which forms an insoluble S-bridging silver(I) complex (Leung *et al.*, 2013 ▸), we have focused on ligand-exchangeable silver(I) complexes with N and O donor atoms. Although many of them are difficult to crystallize and light-sensitive, several crystals of silver(I) complexes have been prepared (Nomiya *et al.*, 2014 ▸). In comparison to gold(I) ions, silver(I) ions show various coord­ination numbers and modes with N and O atoms and tend to form polymeric structures. The polymeric structures of silver(I) complexes with non-S amino acid ligands are classified into four types based on the bonding modes of the silver(I) atom: type I contains only Ag—O bonds, *e.g.*, silver(I) with aspartic acid (Hasp), {[Ag_2_(d-asp)(l-asp)]1.5H_2_O}_*n*_; type II contains O—Ag—O and N—Ag—N bonds, *e.g*., silver(I) with glycine (Hgly), [Ag(gly)]_*n*_; type III contains N—Ag—O units, *e.g.*, silver(I) complexes with glycine, [Ag(gly)]_*n*_, and l-asparagine (l-Hasn), [Ag(l-asn)]_*n*_; type IV contains only Ag—N bonds, *e.g.*, silver(I) with l-histidine (l-H_2_his), [Ag(l-Hhis)]_*n*_ (Nomiya *et al.*, 2000 ▸; Nomiya & Yokoyama, 2002 ▸). Two types of complexes (types II and III) have been reported for [Ag(gly)]_*n*_. Here, we report the preparation and crystal structure of silver(I) with l-valine (l-Hval).
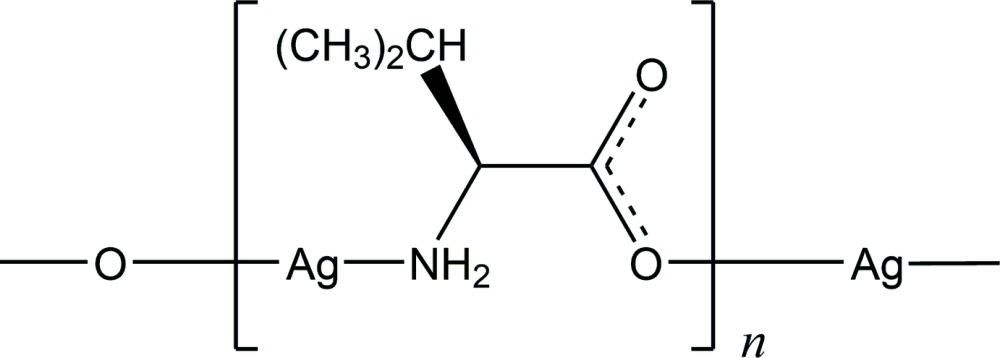


## Structural commentary   

**Figure 1:**
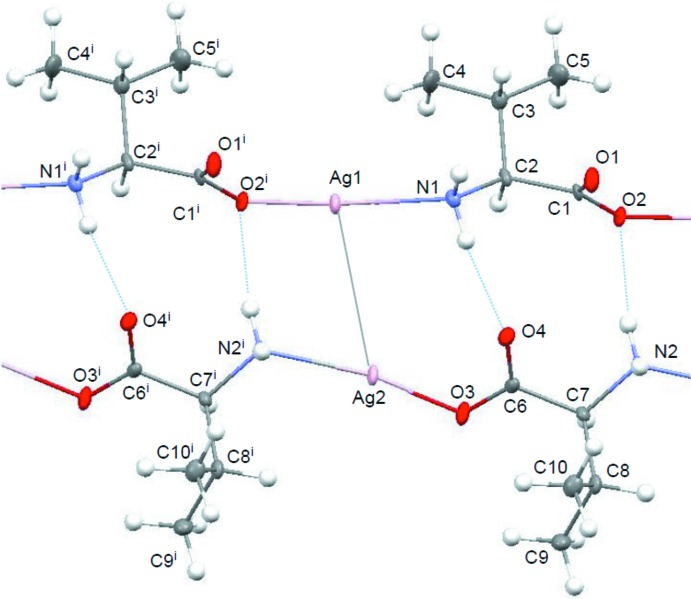
Part of the polymeric structure of the title compound showing the local coordination around the silver(I) atoms. Displacement ellipsoids are drawn at the 50% probability level. The weak Ag⋯Ag inter­action is displayed as a grey line and the N—H⋯O hydrogen bonds are drawn as blue dotted lines. [Symmetry code: (i) *x* − 1, *y*, *z* − 1.]

The local coordination around the silver(I) atom of the title compound is shown in Fig. 1 ▸. The asymmetric unit consists of two units of [Ag(l-val)], which separately form polymeric chains along [101]. In each chain, the N and O atoms coordinate almost linearly to the silver(I) atom (Table 1 ▸), resulting in repeating N—Ag—O units. Since the Ag1⋯O1^iv^ distance [2.654 (4) Å; symmetry code: (iv) *x*, *y*, *z* − 1] is much longer than those of Ag1—O2^i^ [2.124 (3) Å] and Ag2—O3 [2.142 (4) Å], [Ag(l-val)]_*n*_ is classified as being a type III linear N—Ag—O polymer, as found in the silver(I) complexes with glycine (Acland & Freeman, 1971 ▸), with α-alanine (Démaret & Abraham, 1987 ▸) and with asparagine (Nomiya & Yokoyama, 2002 ▸).

Although the polymeric structures of N—Ag—O repeated units of [Ag(l-val)]_*n*_ and [Ag(l-asn)]_*n*_ are similar to each other, the Ag⋯Ag distance [3.3182 (6) Å] between the neighbouring chains in [Ag(l-val)]_*n*_ is slightly shorter than that [3.4371 (9) Å] in [Ag(l-asn)]_*n*_. This indicates the presence of a weak Ag⋯Ag inter­action between the two independent N—Ag—O chains in the title complex, considering the metallic and van der Waals radii of 1.44 and 1.72 Å, respectively, for Ag (Wells, 1975 ▸; Bondi, 1964 ▸).

## Supra­molecular features   

**Figure 2:**
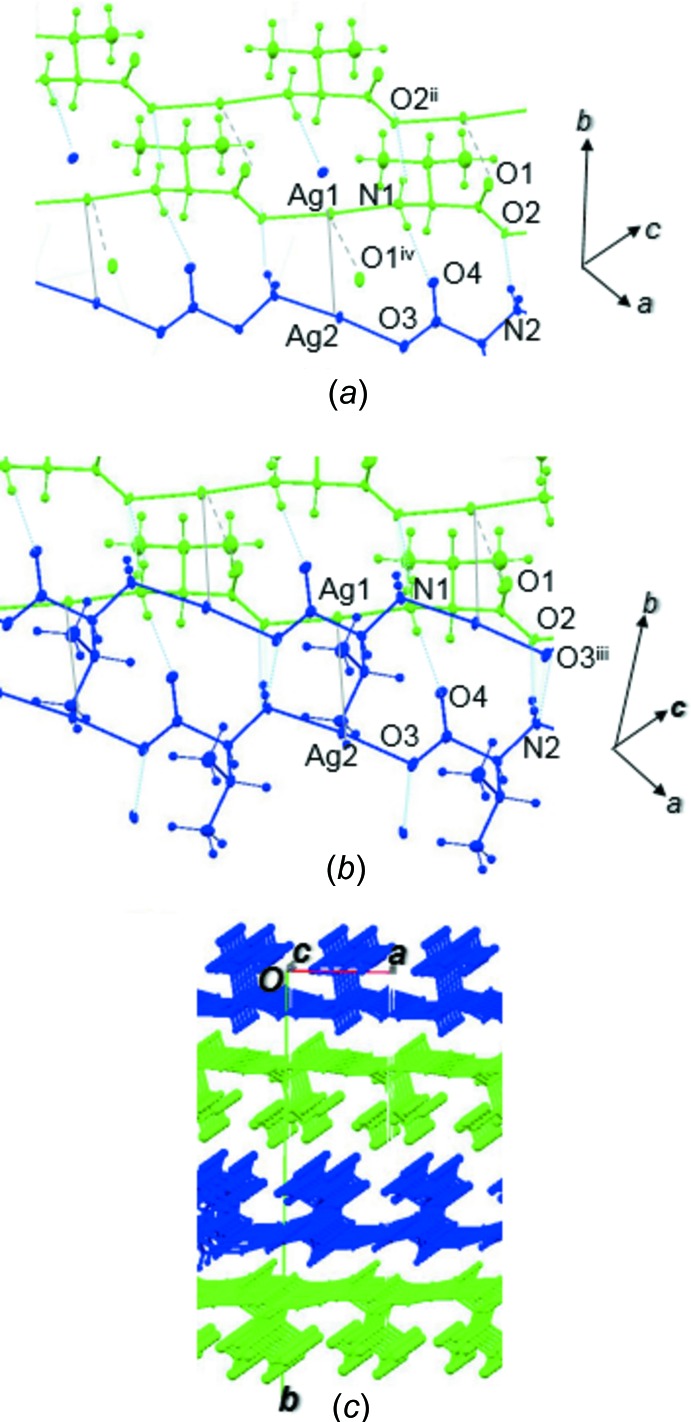
(*a*) Weak inter­actions around the polymeric chains containing Ag1 [symmetry codes: (ii) *x* − 1, *y*, *z*; (iv) *x*, *y*, *z* − 1]. (*b*) Weak inter­actions around the coordination polymers containing Ag2 [symmetry code: (iii) *x*, *y*, *z* + 1]. (*c*) Packing diagram of [Ag(l-val)]_*n*_.

The two independent polymeric chains containing Ag1 and Ag2, respectively, are represented as green and blue in Fig. 2 ▸. The chains of Ag1 are connected to each other by N—H⋯O hydrogen bonds [N1—H1*A*⋯O2^ii^; symmetry code: (ii) *x* − 1, *y*, *z*] into a sheet structure. The chains of Ag2 are also linked into a sheet structure by N—H⋯O hydrogen bonds [N2—H2*A*⋯O3^iii^; symmetry code: (iii) *x*, *y*, *z* + 1]. Both sheets are parallel to the *ac* plane and the two sheets are stacked alternately along the *b* axis through the weak Ag⋯Ag inter­actions and N—H⋯O hydrogen bonds (N1—H1*B*⋯O4 and N2—H22*B*⋯O2; Table 2 ▸).

## Synthesis and crystallization   

To a suspension of 232 mg (1.0 mmol) of Ag_2_O in 20 ml of water was added 234 mg of l-valine (2.0 mmol), followed by stirring for 2 h at room temperature. The resulting grey suspension was filtered. Vapour diffusion was performed at room temperature by using the colourless filtrate as the inner solution and ethanol as the external solvent. The platelet crystals formed were collected and washed with acetone (30 ml) and ether (30 ml) to afford 0.5 mg of colourless crystals of [Ag(l-val)]. The colour of the crystals gradually changed to brown in a few days at ambient temperature. Analysis calculated for C_5_H_10_NO_2_Ag: C 26.81, H 4.50, N 6.25%. Found: C 27.01, H 4.40, N 6.34%. Prominent IR bands in 1800–400 cm^−1^ (KBr disk): 1577*vs*, 1471*m*, 1414*s*, 1359*m*, 1184*w*, 987*w*, 892*w*, 827*m*, 716*m*, 651*m*, 547*m*, 443*m*.

## Anti­microbial activity   

The title silver(I) complex exhibits anti­microbial activity for selected bacteria. The minimum inhibitory concentration (MIC, μ mL^−1^) values of the complex for four bacteria, *E. coli*, *B. subtilis*, *S. aureus*, *P. aeruginosa* are 31.3, 62.5, 125 and 31.3, respectively. [Ag(l-val)]_*n*_ did not inhibit the growth of two yeasts (*C. albicans* and *S. cerevisiae*) and two molds [*A. brasiliensis* (*niger*) and *P. citrinum*] in water-suspension systems. [Ag(l-val)]_*n*_ is insoluble in H_2_O and other organic solvents (MeOH, DMSO, acetone, EtOH, CH_3_CN, CH_2_Cl_2_, CHCl_3_, ether, and EtOAc).

## Refinement   

Crystal data, data collection and structure refinement details are summarized in Table 3 ▸. C-bound H atoms were positioned geometrically and refined using a riding model with *U*_iso_(H) = 1.2 or 1.5*U*_eq_(C). H atoms of the amino groups were found in a difference Fourier map and their positions were refined with restraints of N—H = 0.86 (2) Å and H⋯H = 1.40 (4) Å, and with *U*_iso_(H) = 1.2*U*_eq_(N).

## Supplementary Material

Crystal structure: contains datablock(s) I, global. DOI: 10.1107/S2056989017001815/is5463sup1.cif

Structure factors: contains datablock(s) I. DOI: 10.1107/S2056989017001815/is5463Isup2.hkl

CCDC reference: 1530639

Additional supporting information:  crystallographic information; 3D view; checkCIF report

## Figures and Tables

**Table 1:** Selected geometric parameters (Å, °)

Ag1—N1	2.136 (4)	Ag2—O3	2.142 (4)
Ag1—O2^i^	2.124 (3)	Ag2—N2^i^	2.155 (4)
			
O2^i^—Ag1—N1	176.13 (16)	O3—Ag2—N2^i^	165.79 (18)

Symmetry code: (i) 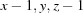.

**Table 2:** Hydrogen-bond geometry (Å, °)

*D*—H⋯*A*	*D*—H	H⋯*A*	*D*⋯*A*	*D*—H⋯*A*
N1—H1*B*⋯O4	0.87 (3)	2.04 (3)	2.907 (6)	169 (5)
N2—H2*B*⋯O2	0.87 (3)	2.19 (3)	3.053 (7)	171 (6)
N1—H1*A*⋯O2^ii^	0.86 (3)	2.10 (3)	2.935 (5)	164 (6)
N2—H2*A*⋯O3^iii^	0.86 (3)	2.13 (4)	2.924 (5)	153 (6)

Symmetry codes: (ii) 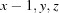; (iii) 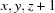.

**Table 3:** Experimental details

Crystal data	
Chemical formula	[Ag(C_5_H_10_NO_2_)]
*M*_r_	224.01
Crystal system, space group	Monoclinic, *P*2_1_
Temperature (K)	90
*a*, *b*, *c* (Å)	5.4475 (5), 22.545 (2), 5.5411 (5)
β (°)	95.446 (2)
*V* (Å^3^)	677.47 (11)
*Z*	4
Radiation type	Mo *K*α
μ (mm^−1^)	2.90
Crystal size (mm)	0.36 × 0.16 × 0.09

Data collection	
Diffractometer	Bruker SMART APEXII CCD
Absorption correction	Multi-scan (*SADABS*; Bruker, 2009 ▸)
*T*_min_, *T*_max_	0.422, 0.780
No. of measured, independent and observed [*I* > 2σ(*I*)] reflections	4981, 3034, 3013
*R*_int_	0.016
(sin θ/λ)_max_ (Å^−1^)	0.666

Refinement	
*R*[*F* ^2^ > 2σ(*F* ^2^)], *wR*(*F* ^2^), *S*	0.022, 0.054, 1.15
No. of reflections	3034
No. of parameters	179
No. of restraints	7
H-atom treatment	H atoms treated by a mixture of independent and constrained refinement
Δρ_max_, Δρ_min_ (e Å^−3^)	1.13, −1.11
Absolute structure	Flack *x* determined using 1275 quotients [(*I* ^+^)−(*I* ^−^)]/[(*I* ^+^)+(*I* ^−^)] (Parsons *et al.*, 2013 ▸)
Absolute structure parameter	0.048 (19)

Computer programs: *APEX2* and *SAINT* (Bruker, 2008 ▸), *SIR2004* (Burla *et al.*, 2005 ▸), *SHELXL2016* (Sheldrick, 2015 ▸) and *Mercury* (Macrae *et al.*, 2008 ▸).

## supplementary crystallographic information

### Crystal data

**Table:**

[Ag(C_5_H_10_NO_2_)]	*F*(000) = 440
*M**_r_* = 224.01	*D*_x_ = 2.196 Mg m^−^^3^
Monoclinic, *P*2_1_	Mo *K*α radiation, λ = 0.71073 Å
*a* = 5.4475 (5) Å	Cell parameters from 4323 reflections
*b* = 22.545 (2) Å	θ = 3.6–28.3°
*c* = 5.5411 (5) Å	µ = 2.90 mm^−^^1^
β = 95.446 (2)°	*T* = 90 K
*V* = 677.47 (11) Å^3^	Needle, colorless
*Z* = 4	0.36 × 0.16 × 0.09 mm

### Data collection

**Table:**

Bruker SMART APEXII CCD diffractometer	3034 independent reflections
Radiation source: Sealed Tube	3013 reflections with *I* > 2σ(*I*)
Detector resolution: 8.366 pixels mm^-1^	*R*_int_ = 0.016
ω scans	θ_max_ = 28.3°, θ_min_ = 1.8°
Absorption correction: multi-scan (SADABS; Bruker, 2009)	*h* = −7→3
*T*_min_ = 0.422, *T*_max_ = 0.780	*k* = −28→30
4981 measured reflections	*l* = −7→7

### Refinement

**Table:**

Refinement on *F*^2^	H atoms treated by a mixture of independent and constrained refinement
Least-squares matrix: full	*w* = 1/[σ^2^(*F*_o_^2^) + (0.0258*P*)^2^ + 0.4276*P*] where *P* = (*F*_o_^2^ + 2*F*_c_^2^)/3
*R*[*F*^2^ > 2σ(*F*^2^)] = 0.022	(Δ/σ)_max_ = 0.001
*wR*(*F*^2^) = 0.054	Δρ_max_ = 1.13 e Å^−^^3^
*S* = 1.15	Δρ_min_ = −1.11 e Å^−^^3^
3034 reflections	Absolute structure: Flack *x* determined using 1275 quotients [(*I*^+^)-(*I*^-^)]/[(*I*^+^)+(*I*^-^)] (Parsons *et al.*, 2013)
179 parameters	Absolute structure parameter: 0.048 (19)
7 restraints	

### Special details

**Table:**

Geometry. All esds (except the esd in the dihedral angle between two l.s. planes) are estimated using the full covariance matrix. The cell esds are taken into account individually in the estimation of esds in distances, angles and torsion angles; correlations between esds in cell parameters are only used when they are defined by crystal symmetry. An approximate (isotropic) treatment of cell esds is used for estimating esds involving l.s. planes.
Refinement. Refinement of F^2^ against ALL reflections. The weighted R-factor wR and goodness of fit S are based on F^2^, conventional R-factors R are based on F, with F set to zero for negative F^2^. The threshold expression of F^2^ > 2sigma(F^2^) is used only for calculating R-factors(gt) etc. and is not relevant to the choice of reflections for refinement. R-factors based on F^2^ are statistically about twice as large as those based on F, and R- factors based on ALL data will be even larger.

### Fractional atomic coordinates and isotropic or equivalent isotropic displacement parameters (Å^2^)

**Table:**

	*x*	*y*	*z*	*U*_iso_*/*U*_eq_	
Ag1	0.62135 (6)	0.72712 (2)	0.36519 (6)	0.01288 (9)	
C1	1.1756 (9)	0.7379 (2)	1.0188 (9)	0.0097 (10)	
C2	1.0368 (9)	0.7590 (2)	0.7813 (9)	0.0112 (9)	
H2C	1.149003	0.755936	0.649045	0.013*	
C3	0.9626 (9)	0.8245 (2)	0.8085 (10)	0.0146 (10)	
H3	0.837026	0.826310	0.928715	0.018*	
C4	0.8460 (11)	0.8514 (3)	0.5695 (11)	0.0244 (12)	
H4A	0.962402	0.848370	0.445617	0.037*	
H4B	0.806760	0.893266	0.594925	0.037*	
H4C	0.694559	0.829836	0.515562	0.037*	
C5	1.1823 (11)	0.8626 (3)	0.9056 (12)	0.0236 (12)	
H5A	1.309746	0.861056	0.792249	0.035*	
H5B	1.249657	0.847404	1.063895	0.035*	
H5C	1.128339	0.903713	0.922864	0.035*	
O1	1.0588 (6)	0.7234 (2)	1.1919 (6)	0.0166 (7)	
O2	1.4109 (6)	0.73698 (16)	1.0246 (6)	0.0125 (7)	
N1	0.8165 (8)	0.7214 (2)	0.7175 (7)	0.0115 (8)	
H1A	0.721 (9)	0.726 (3)	0.830 (8)	0.014*	
H1B	0.877 (10)	0.6856 (15)	0.732 (11)	0.014*	
Ag2	0.89815 (6)	0.60339 (2)	0.21275 (6)	0.01329 (10)	
C6	1.2547 (9)	0.5896 (2)	0.6450 (10)	0.0107 (9)	
C7	1.5054 (9)	0.5750 (2)	0.7793 (9)	0.0102 (9)	
H7	1.635708	0.590634	0.680772	0.012*	
C8	1.5466 (9)	0.5077 (2)	0.8113 (9)	0.0120 (9)	
H8	1.686570	0.502380	0.939835	0.014*	
C9	1.6241 (10)	0.4791 (3)	0.5799 (10)	0.0185 (10)	
H9A	1.487275	0.481332	0.451923	0.028*	
H9B	1.667499	0.437482	0.611812	0.028*	
H9C	1.767197	0.500216	0.527632	0.028*	
C10	1.3225 (10)	0.4764 (2)	0.8998 (10)	0.0168 (10)	
H10A	1.365825	0.435354	0.943053	0.025*	
H10B	1.186513	0.476776	0.770720	0.025*	
H10C	1.271796	0.497164	1.042428	0.025*	
N2	1.5353 (7)	0.6050 (2)	1.0205 (7)	0.0118 (7)	
H2A	1.442 (10)	0.587 (2)	1.112 (10)	0.014*	
H2B	1.494 (11)	0.6423 (13)	1.004 (11)	0.014*	
O3	1.2360 (7)	0.57934 (18)	0.4188 (7)	0.0154 (8)	
O4	1.0857 (6)	0.61000 (18)	0.7610 (6)	0.0140 (7)	

### Atomic displacement parameters (Å^2^)

**Table:**

	*U*^11^	*U*^22^	*U*^33^	*U*^12^	*U*^13^	*U*^23^
Ag1	0.00939 (17)	0.01968 (19)	0.00880 (17)	0.00032 (14)	−0.00310 (11)	−0.00117 (15)
C1	0.010 (2)	0.012 (3)	0.007 (2)	−0.0023 (17)	−0.0027 (17)	−0.0017 (17)
C2	0.008 (2)	0.017 (3)	0.008 (2)	−0.0015 (18)	−0.0004 (17)	−0.0040 (18)
C3	0.013 (2)	0.015 (2)	0.016 (2)	−0.0009 (18)	−0.0019 (19)	−0.0001 (19)
C4	0.031 (3)	0.020 (3)	0.020 (3)	0.006 (3)	−0.012 (2)	0.004 (2)
C5	0.024 (3)	0.016 (3)	0.029 (3)	−0.003 (2)	−0.006 (2)	−0.002 (2)
O1	0.0121 (16)	0.027 (2)	0.0102 (16)	−0.0014 (16)	−0.0009 (12)	0.0029 (17)
O2	0.0035 (14)	0.021 (2)	0.0123 (16)	0.0017 (12)	−0.0014 (12)	−0.0017 (14)
N1	0.0099 (18)	0.014 (2)	0.0104 (18)	−0.0014 (16)	0.0010 (14)	−0.0020 (17)
Ag2	0.00789 (17)	0.0215 (2)	0.00975 (18)	0.00137 (14)	−0.00313 (12)	−0.00090 (15)
C6	0.009 (2)	0.008 (2)	0.015 (2)	−0.0007 (16)	−0.0017 (18)	0.0003 (16)
C7	0.010 (2)	0.012 (2)	0.008 (2)	0.0006 (17)	0.0005 (17)	−0.0026 (17)
C8	0.008 (2)	0.017 (2)	0.011 (2)	−0.0003 (18)	−0.0017 (17)	−0.0003 (18)
C9	0.019 (3)	0.019 (3)	0.017 (2)	0.007 (2)	0.000 (2)	−0.004 (2)
C10	0.014 (2)	0.018 (2)	0.019 (3)	−0.002 (2)	0.002 (2)	0.004 (2)
N2	0.0130 (19)	0.0164 (19)	0.0054 (18)	0.0006 (18)	−0.0028 (14)	0.0000 (18)
O3	0.0088 (16)	0.027 (2)	0.0099 (17)	0.0028 (14)	−0.0009 (13)	0.0020 (15)
O4	0.0092 (15)	0.0187 (19)	0.0142 (18)	0.0034 (14)	0.0006 (13)	−0.0009 (15)

### Geometric parameters (Å, º)

**Table:**

Ag1—N1	2.136 (4)	Ag2—O3	2.142 (4)
Ag1—O2^i^	2.124 (3)	Ag2—N2^i^	2.155 (4)
C1—O1	1.245 (6)	C6—O4	1.259 (6)
C1—O2	1.280 (6)	C6—O3	1.269 (7)
C1—C2	1.530 (7)	C6—C7	1.527 (7)
C2—N1	1.485 (6)	C7—N2	1.493 (6)
C2—C3	1.543 (7)	C7—C8	1.541 (7)
C2—H2C	1.0000	C7—H7	1.0000
C3—C5	1.528 (8)	C8—C10	1.530 (7)
C3—C4	1.538 (7)	C8—C9	1.530 (7)
C3—H3	1.0000	C8—H8	1.0000
C4—H4A	0.9800	C9—H9A	0.9800
C4—H4B	0.9800	C9—H9B	0.9800
C4—H4C	0.9800	C9—H9C	0.9800
C5—H5A	0.9800	C10—H10A	0.9800
C5—H5B	0.9800	C10—H10B	0.9800
C5—H5C	0.9800	C10—H10C	0.9800
N1—H1A	0.86 (3)	N2—H2A	0.86 (3)
N1—H1B	0.87 (3)	N2—H2B	0.87 (3)
			
O2^i^—Ag1—N1	176.13 (16)	O3—Ag2—N2^i^	165.79 (18)
O1—C1—O2	124.1 (5)	O4—C6—O3	125.2 (5)
O1—C1—C2	119.9 (4)	O4—C6—C7	119.5 (5)
O2—C1—C2	116.0 (4)	O3—C6—C7	115.2 (4)
N1—C2—C1	110.4 (4)	N2—C7—C6	110.8 (4)
N1—C2—C3	110.9 (4)	N2—C7—C8	109.9 (4)
C1—C2—C3	109.1 (4)	C6—C7—C8	112.4 (4)
N1—C2—H2C	108.8	N2—C7—H7	107.8
C1—C2—H2C	108.8	C6—C7—H7	107.8
C3—C2—H2C	108.8	C8—C7—H7	107.8
C5—C3—C4	109.1 (5)	C10—C8—C9	111.5 (4)
C5—C3—C2	111.6 (4)	C10—C8—C7	112.2 (4)
C4—C3—C2	112.6 (5)	C9—C8—C7	111.5 (4)
C5—C3—H3	107.8	C10—C8—H8	107.1
C4—C3—H3	107.8	C9—C8—H8	107.1
C2—C3—H3	107.8	C7—C8—H8	107.1
C3—C4—H4A	109.5	C8—C9—H9A	109.5
C3—C4—H4B	109.5	C8—C9—H9B	109.5
H4A—C4—H4B	109.5	H9A—C9—H9B	109.5
C3—C4—H4C	109.5	C8—C9—H9C	109.5
H4A—C4—H4C	109.5	H9A—C9—H9C	109.5
H4B—C4—H4C	109.5	H9B—C9—H9C	109.5
C3—C5—H5A	109.5	C8—C10—H10A	109.5
C3—C5—H5B	109.5	C8—C10—H10B	109.5
H5A—C5—H5B	109.5	H10A—C10—H10B	109.5
C3—C5—H5C	109.5	C8—C10—H10C	109.5
H5A—C5—H5C	109.5	H10A—C10—H10C	109.5
H5B—C5—H5C	109.5	H10B—C10—H10C	109.5
C2—N1—Ag1	120.3 (3)	C7—N2—H2A	107 (4)
C2—N1—H1A	107 (4)	C7—N2—H2B	110 (4)
C2—N1—H1B	102 (4)	H2A—N2—H2B	112 (5)
H1A—N1—H1B	108 (5)	C6—O3—Ag2	117.7 (3)

Symmetry code: (i) *x*−1, *y*, *z*−1.

### Hydrogen-bond geometry (Å, º)

**Table:**

*D*—H···*A*	*D*—H	H···*A*	*D*···*A*	*D*—H···*A*
N1—H1*B*···O4	0.87 (3)	2.04 (3)	2.907 (6)	169 (5)
N2—H2*B*···O2	0.87 (3)	2.19 (3)	3.053 (7)	171 (6)
N1—H1*A*···O2^ii^	0.86 (3)	2.10 (3)	2.935 (5)	164 (6)
N2—H2*A*···O3^iii^	0.86 (3)	2.13 (4)	2.924 (5)	153 (6)

Symmetry codes: (ii) *x*−1, *y*, *z*; (iii) *x*, *y*, *z*+1.
